# Supplementary material for: USP53 Exerts Tumor-Promoting Effects in Triple-Negative Breast Cancer by Deubiquitinating CRKL
Source: Cancers (Basel). 2023 Oct 18;15(20):5033. doi: 10.3390/cancers15205033 (PMC10605207; doi:10.3390/cancers15205033)

Figure 1a

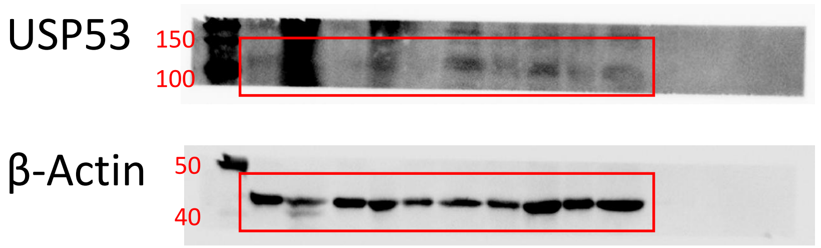

Figure 1i

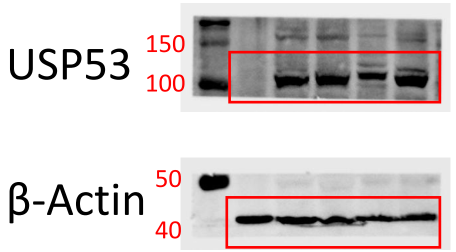

Figure 2a

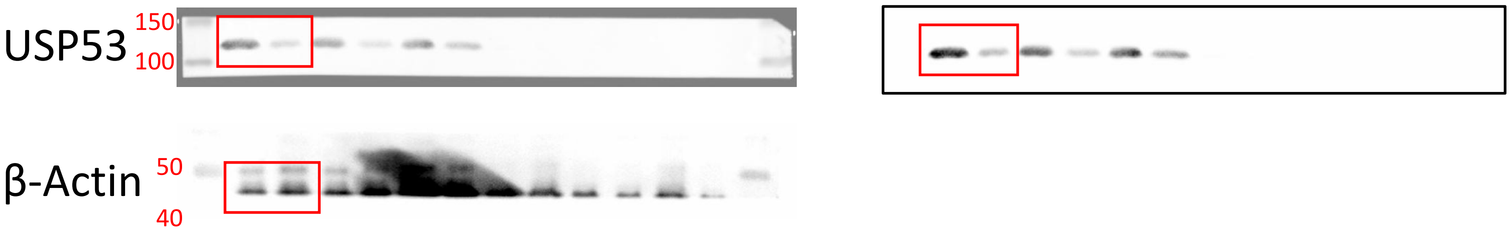

Figure 2d

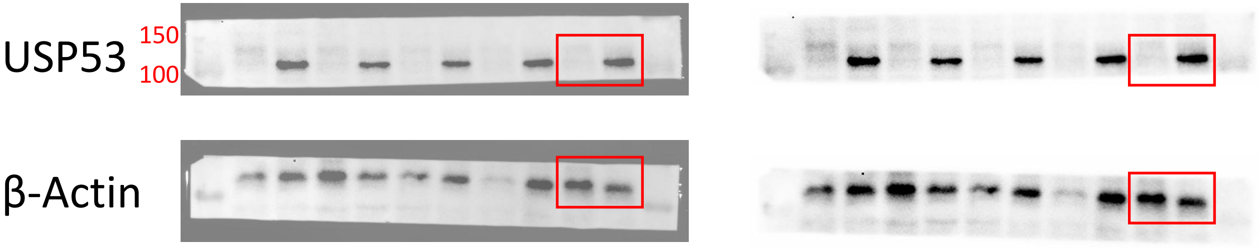

Figure 2i

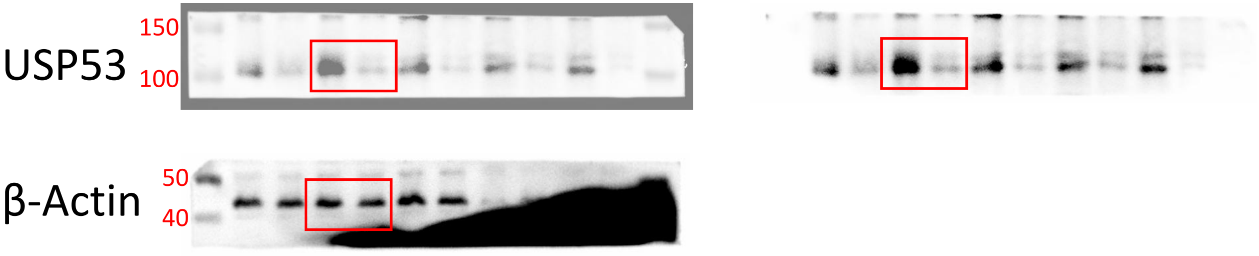

Figure 3e

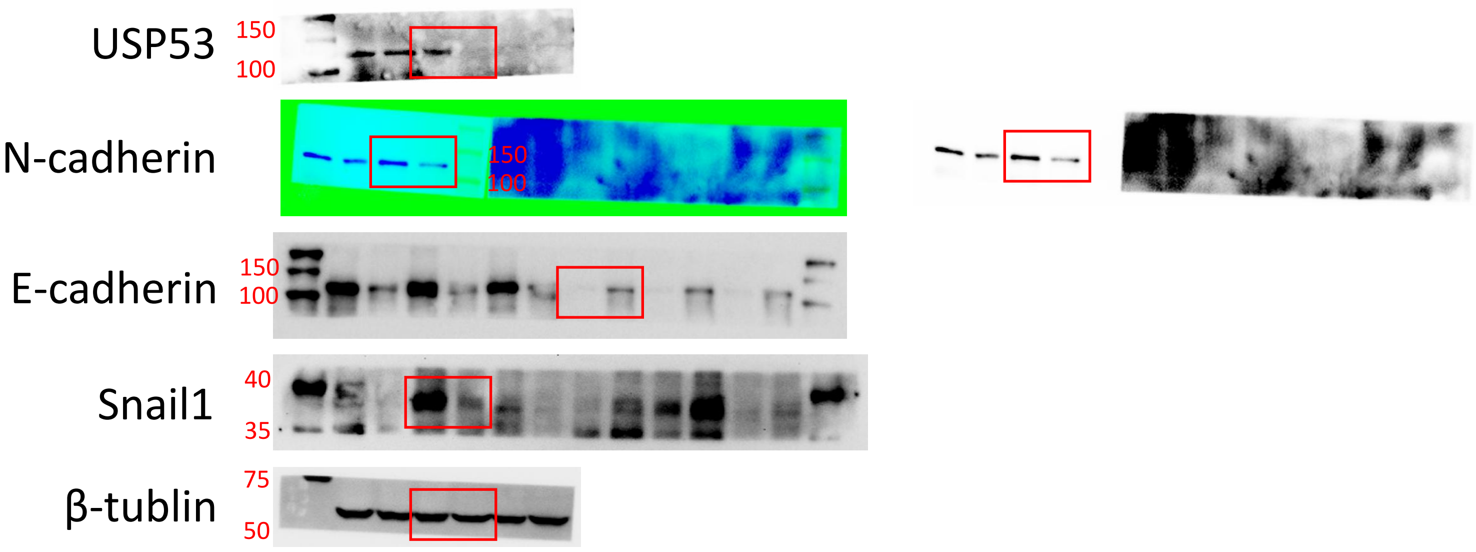

Figure 3g

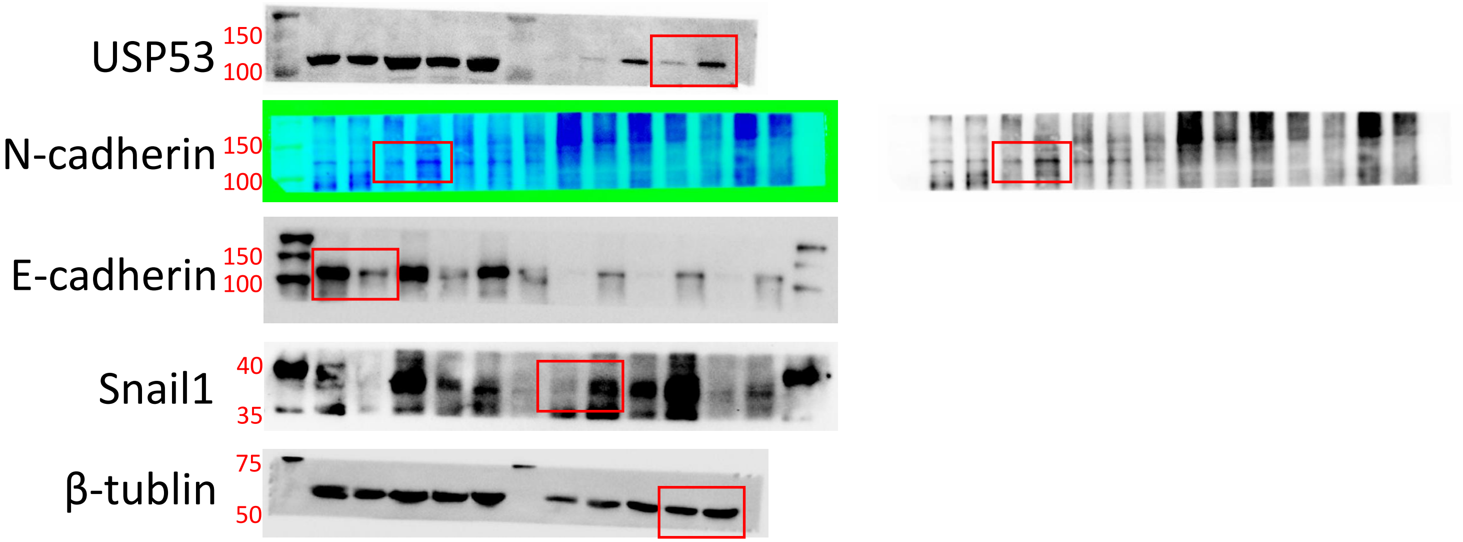

Figure 4b

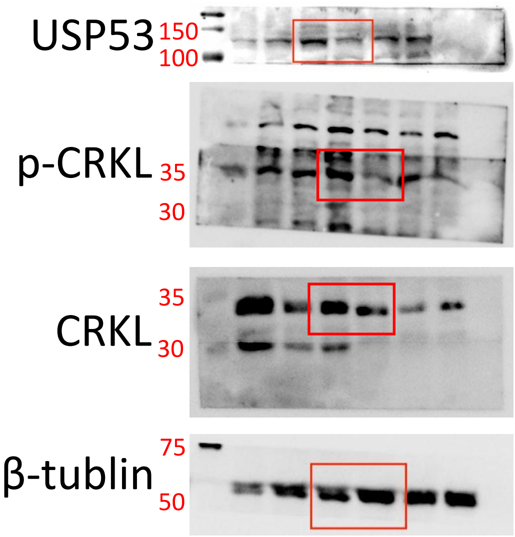

Figure 4d

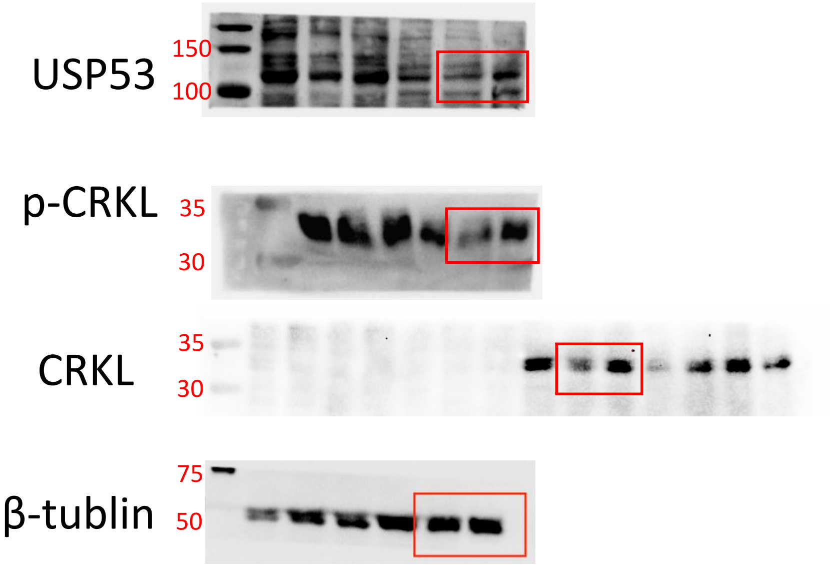

Figure 5c

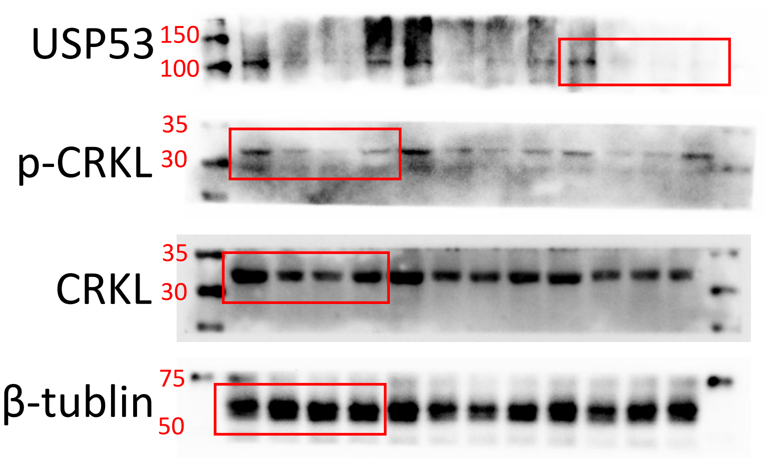

Figure 5e

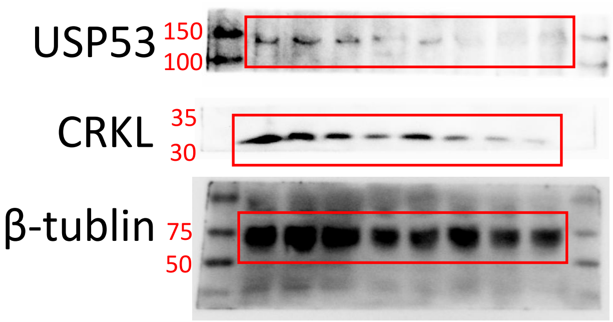

Figure 5g

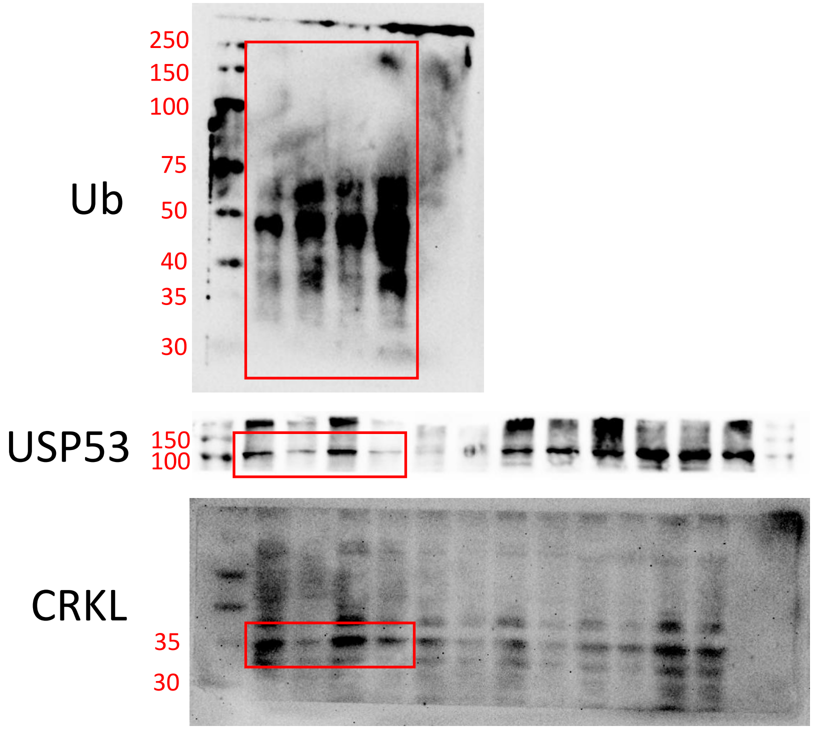

Figure 5h

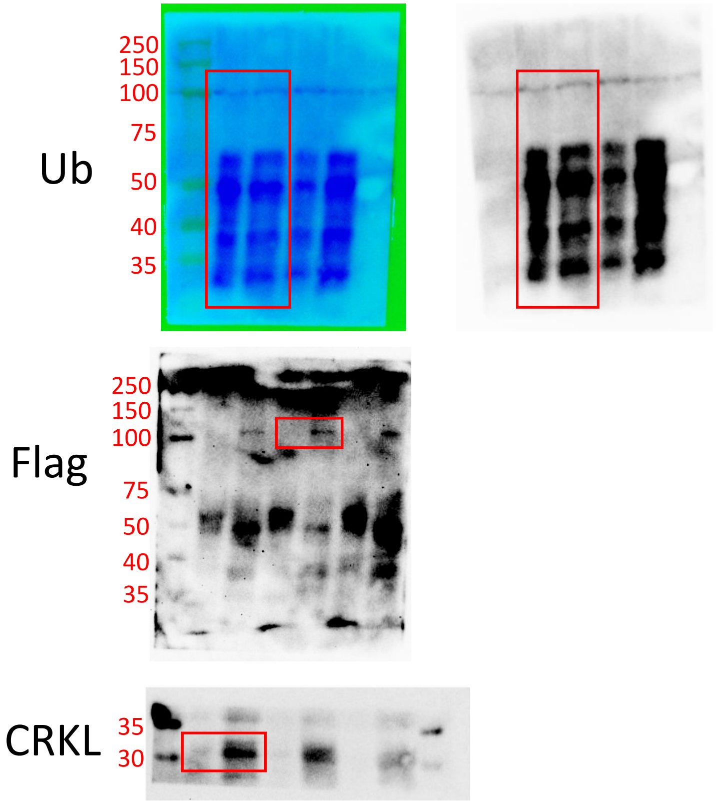

Figure 5i

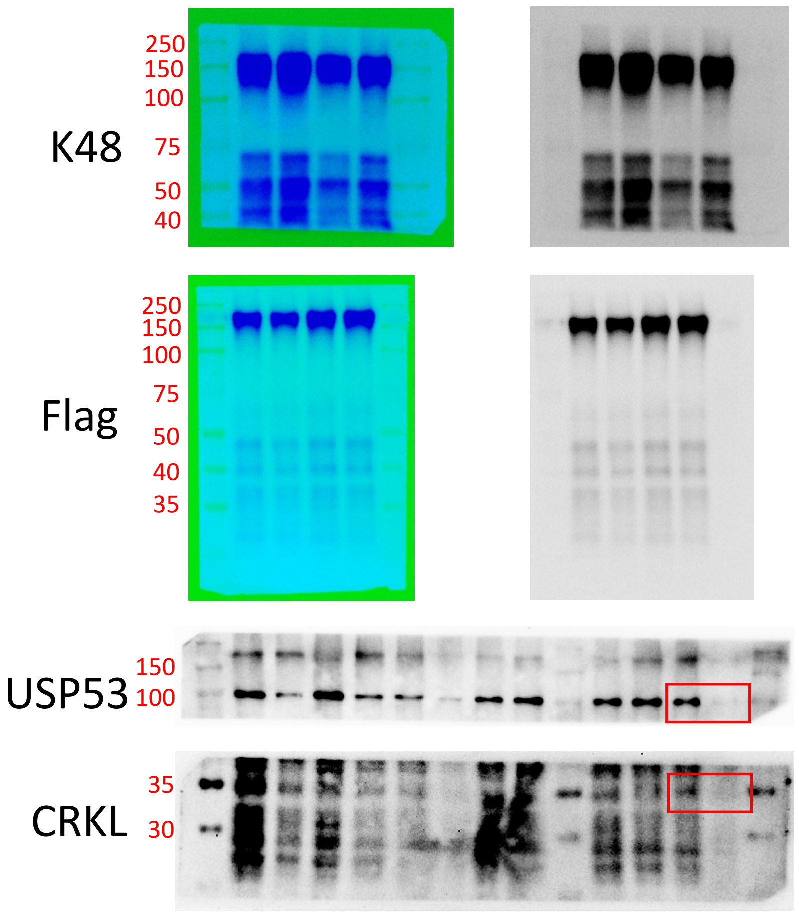
K48
250
150
100
75
50
40
Flag
250
150
100
75
50
40
35
USP53
150
100
CRKL
35
30

Figure 5j

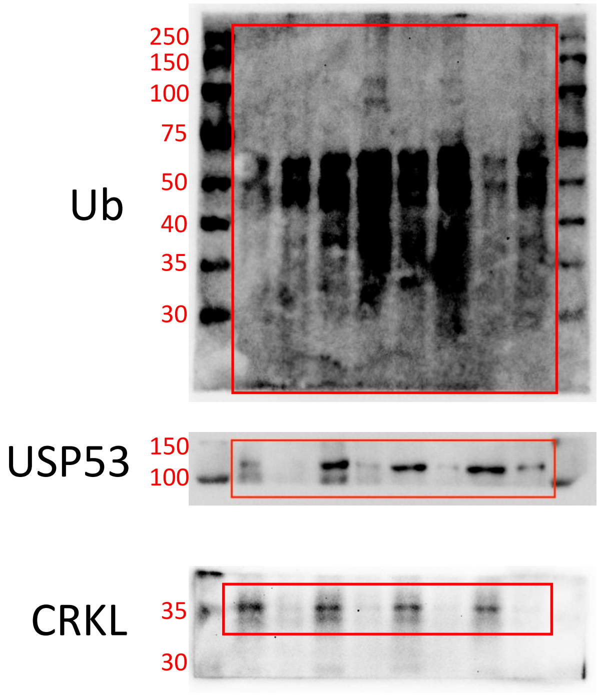

Figure 5l

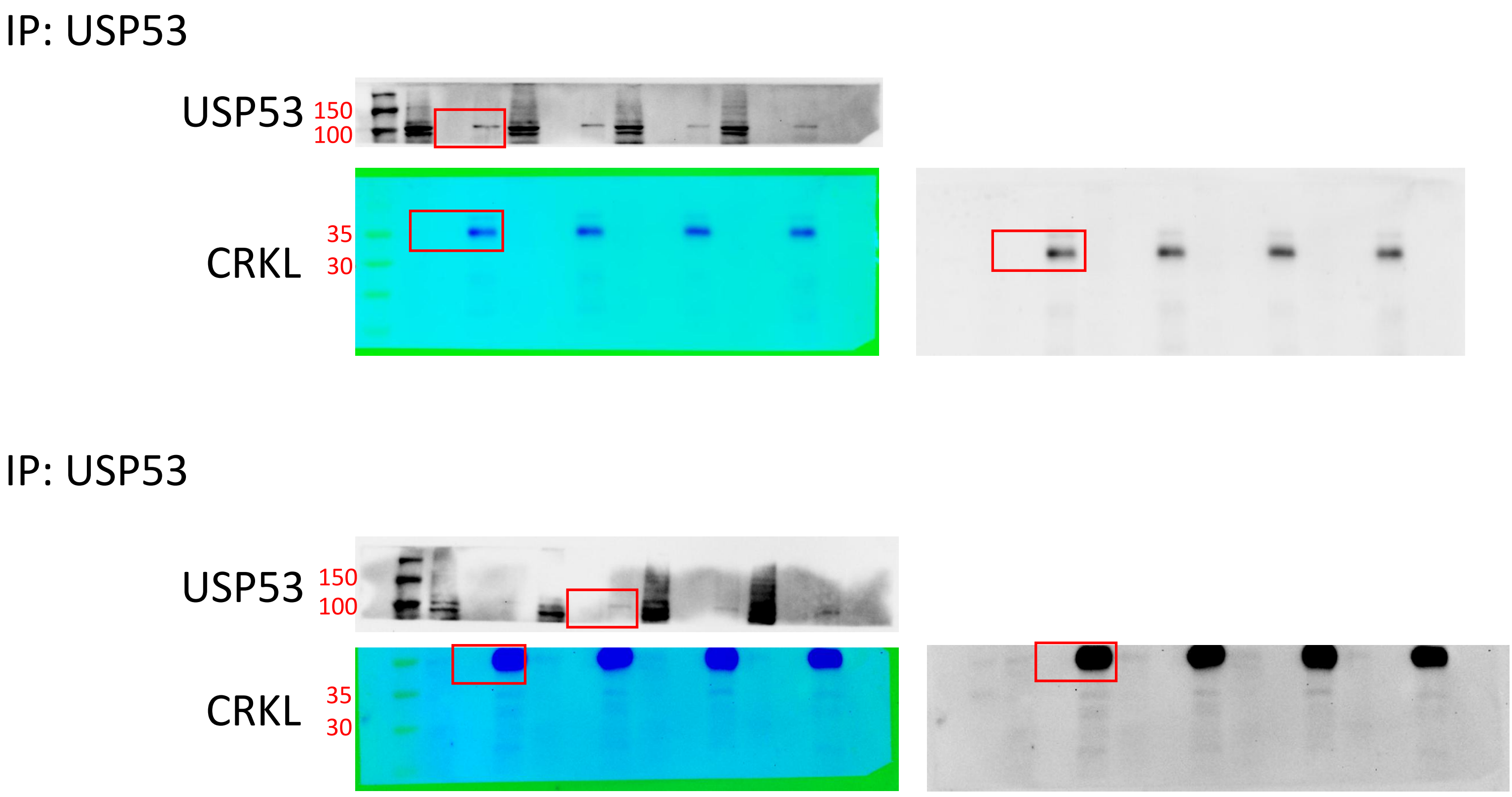
IP: USP53
USP53
150
100
CRKL
35
30
IP: USP53
USP53
150
100
CRKL
35
30

Figure 5m

IP: USP53

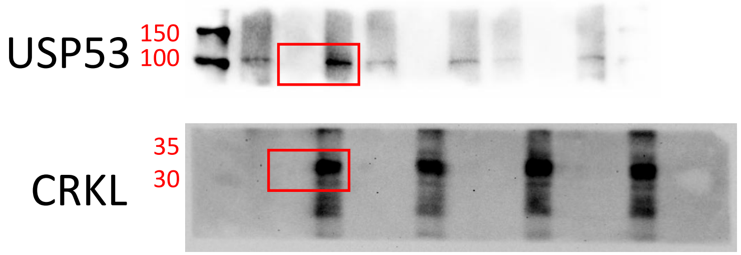

IP: USP53

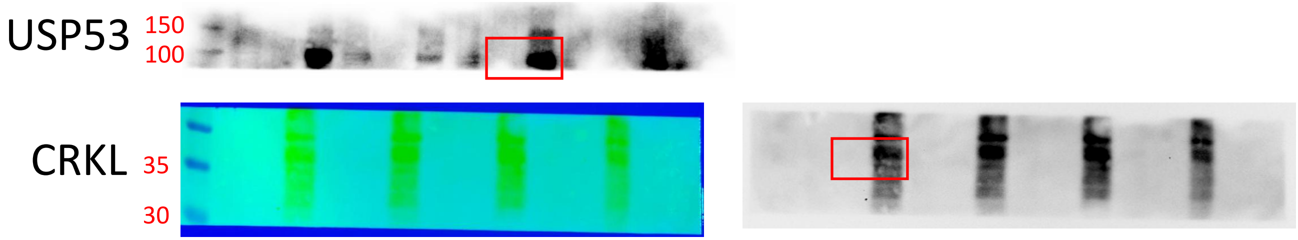

Figure 6a

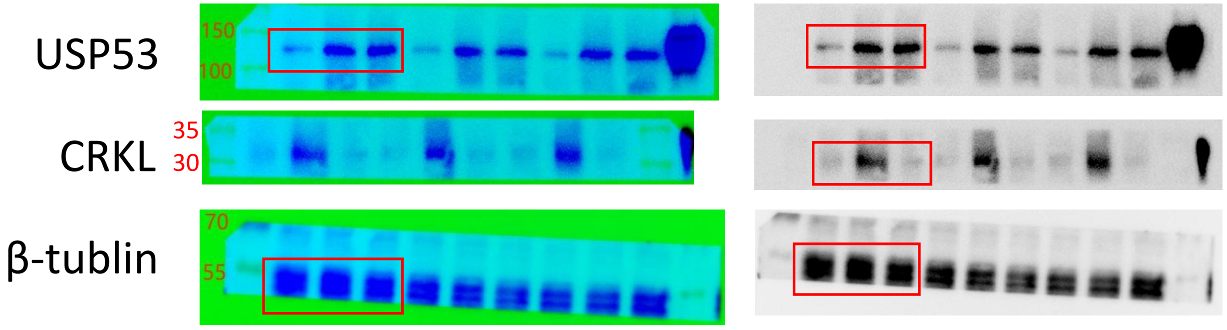

Figure 6g

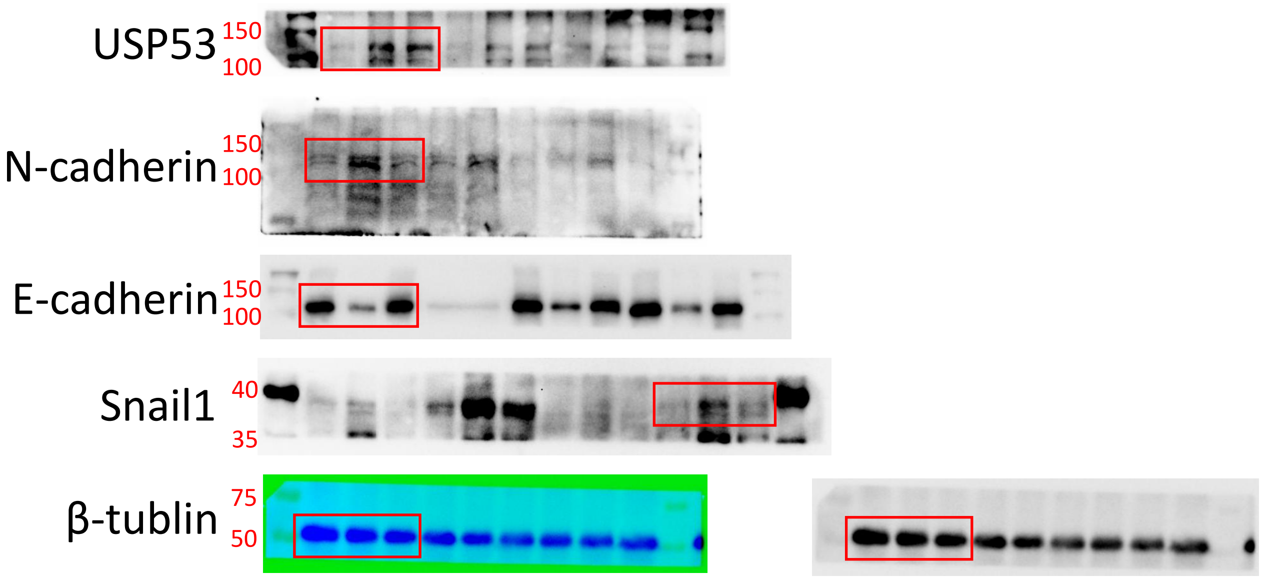

Figure 6h

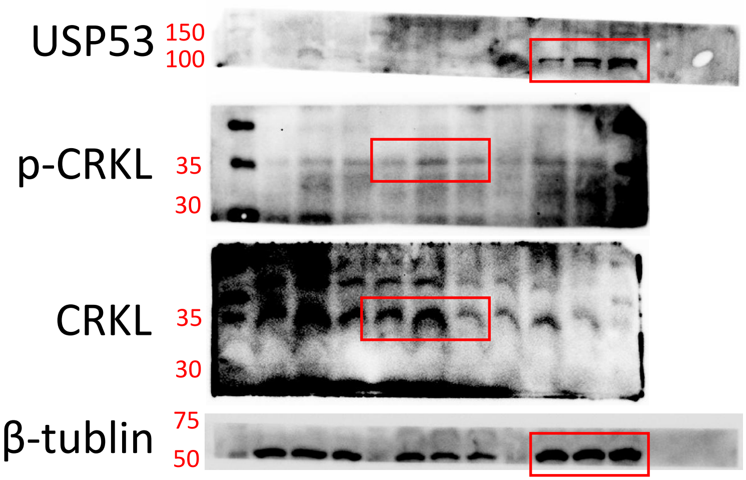

Figure 7a

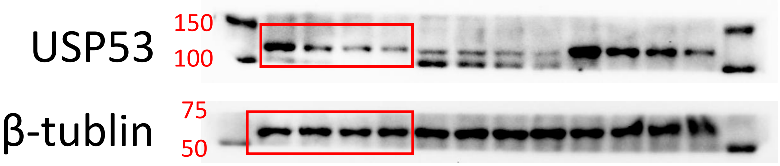

Figure 7b

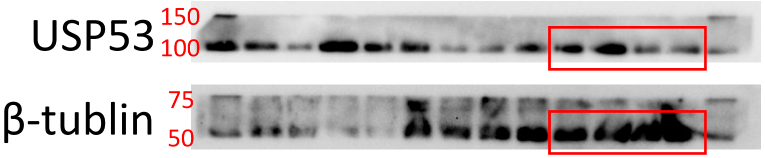

Figure 7k

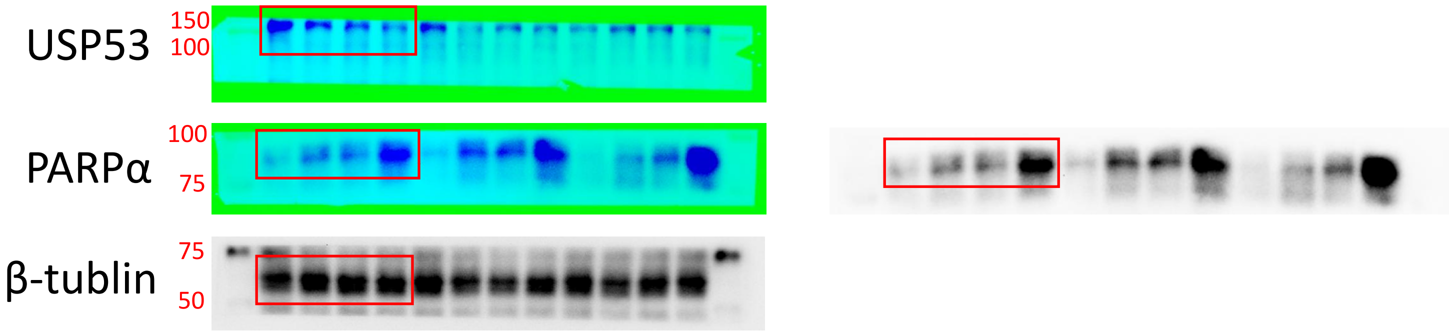

Figure 7l

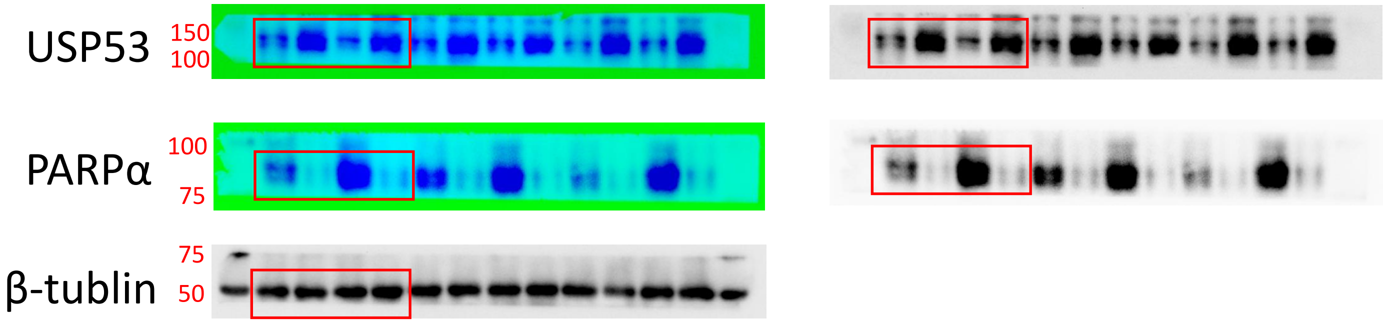

Supplement: Supplementary file 1 [file cancers-15-05033-s001.zip › File S1-Original western blots.pdf]
